# Repulsive Forces Between Looping Chromosomes Induce Entropy-Driven Segregation

**DOI:** 10.1371/journal.pone.0014428

**Published:** 2011-01-04

**Authors:** Manfred Bohn, Dieter W. Heermann

**Affiliations:** Institute for Theoretical Physics, Heidelberg University, Heidelberg, Germany; University of Milano-Bicocca, Italy

## Abstract

One striking feature of chromatin organization is that chromosomes are compartmentalized into distinct territories during interphase, the degree of intermingling being much smaller than expected for linear chains. A growing body of evidence indicates that the formation of loops plays a dominant role in transcriptional regulation as well as the entropic organization of interphase chromosomes. Using a recently proposed model, we quantitatively determine the entropic forces between chromosomes. This Dynamic Loop Model assumes that loops form solely on the basis of diffusional motion without invoking other long-range interactions. We find that introducing loops into the structure of chromatin results in a multi-fold higher repulsion between chromosomes compared to linear chains. Strong effects are observed for the tendency of a non-random alignment; the overlap volume between chromosomes decays fast with increasing loop number. Our results suggest that the formation of chromatin loops imposes both compartmentalization as well as order on the system without requiring additional energy-consuming processes.

## Introduction

Chromatin in higher eukaryotes is compacted and folded on several scales. While the folding mechanisms on the scale of the chromatin fiber are rather well understood [1], [2], the higher-order arrangement of whole chromosomes inside the nucleus remains an open question [3]–[5]. Chromosomes, in comparison to other polymeric systems, display a vast amount of unexpected types of behaviour. Most importantly, chromosomes are highly compartmentalized objects being well separated during interphase [6]. Such a separation is not only observed regarding complete chromosomes, rather Mb-sized stretches of chromatin, when labelled fluorescently with different colors, also display little intermingling [7]. A lot of speculation has been going on about the mechanisms driving such kind of compartmentalization. It has been proposed that compartmentalization results from non-equilibrium effects: During metaphase chromosomes are condensed and well separated. The entanglement time disregarding topoisomerase-II activity is supposed to be much larger than the lifetime of the cell [8]. Based on the assumption of a linear polymer model, calculations show that this holds also true given the activity of topoisomerase-II [9].

Nowadays however, there is an ever growing body of evidence that chromatin is not organized as a simple linear polymer. Rather functional loops seem to play a pivotal role in transcriptional regulation of higher eukaryotes. Several experimental techniques have allowed the determination of specific intra- as well as interchromosomal contacts [10]–[12]. 4C experiments revealed that abundant short as well as long-range chromosomal contacts are established ranging from a few kb to several Mb, these contacts being cell-type specific. The formation of chromatin loops has been shown to be important for transcriptional regulation in the 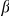-globin locus [13]. Experimental evidence further suggests that loop formation involves specific proteins like CTCF [14].

Recently, several computational studies have been put forward investigating the entropic effects of looping. It was shown that ring polymers in proximity show less intermingling than corresponding linear systems [15]. Cook *et al.* [16] simulated linear chains as well as rosette-like structures in a dense system and found that the probability of inter-chain contacts decreases by the transition from a linear to a looping polymer. In another study, the potential of mean force arising when two ring polymers are brought in proximity has been analyzed [17]. A three-fold increase in the repulsion compared to linear chains (self-avoiding walks) at short separations was found. Topological constraints arising from the non-catenation of rings results in a further increase of repulsion. However, it remains an open question, whether similar predictions can be derived for a system of chromosomes.

The scope of this paper is to extend the study of effective interactions between ring polymers to the more complex system of looping chromosomes. Chromsomes are simulated using the Dynamic Loop (DL) model [18], which has been shown to be consistent with a variety of experimental observation. Among others, the model correctly displays the observed folding into a confined sub-space of the nucleus [19], the power-law decay of the contact probabilities determined in a recent study [12] and the formation of chromosome territories (CTs). This DL model assumes that loops form on the basis of diffusional motion, two chromatin segments having a certain probability to stick together for a certain time when being in proximity. Amongst others, we want to investigate how the strength of the repulsive interaction changes when introducing more and more loops into the system. While a coarse-grained model of the chromosome is employed here, a mapping to physical units can be conducted using results from fluorescence in situ hybridization (FISH) experiments [19].

## Results

### Simulation of chromosomes

To obtain information about the repulsive interactions between chromosomes, we use the recently developed Dynamic Loop (DL) model. This model initally assumes chromatin to consist of a coarse-grained linear polymer chain. Loop formation is achieved using diffusional motion of the monomers in the following way: Whenever two segments co-localize by diffusional motion, a chromatin loop is formed with a certain probability 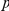 between these two sites. The loop is assigned a certain lifetime, thus loop attachment points dissolve again during the course of time.

The stochastic nature of loop formation provides a method to effectively incorporate protein-chromatin and chromatin-chromatin interactions. Probabilistic looping, which is often thought to be mediated by DNA-binding factors [14], [20], [21] or by transcription factories [22], mimics the effect of protein concentration (there being either proteins binding DNA sites or not) and binding affinity. In the following we denote by “loop” a functional interaction between two parts of a chromatin fiber existing for a certain time as created by the method. In contrast, a “contact” denotes two parts of the chromatin fiber close together by thermal fluctuations without necessarily being an interaction.

A typical human chromosome has a length of about 100 mega basepairs (Mb), rendering a detailed description on the molecular level computationally impossible. Coarse-graining approaches, where a long stretch of chromatin is modeled as an effective monomer, are well justified on a scale above the persistence length 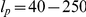 nm [23]. Thus, it is reasonable to conduct computer simulations on a coarse-grained scale where it can be securely assumed that the fiber is flexible.

### The effective repulsion between chromosomes increases strongly with loop number

**Figure 1:**
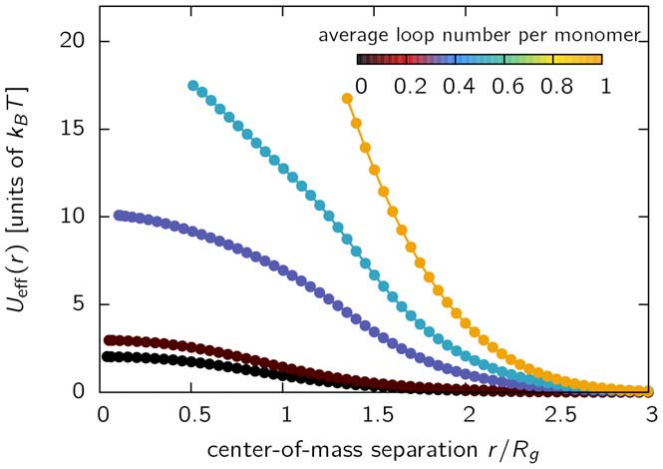
The effective potential 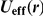 between the centers of mass of looping polymers. Simulations have been conducted using the DL model for chain length 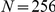 and different looping probabilities 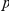. The average number of loops per monomer resulting from the parameter 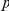 is indicated by the color bar. Data is scaled with the radius of gyration of isolated polymer chains to allow comparison between different parameters sets. The effective potential increases strongly with looping probability 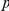. In Figure S1 we furthermore find that the order of magnitude of repulsive interactions is independent of chain length 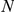 in the range studied.

What happens, when two polymeric coils are brought close together? Clearly, in the absence of other interactions than excluded volume forces, polymers repell each other due to the constrained conformational space available. Such a behaviour has been found both for linear self-avoiding walks [24] and ring polymers [17]. Here, we investigate the potential of mean force between the centers of mass of two chromosomes modelled by the Dynamic Loop model. Results are shown for chains of length 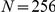 in Figure 1. In principle, dependent on the coarse-graining used, such chains could represent small chromosomal regions up to whole chromosomes. To allow comparison for different sets of parameters (chain length 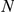, looping probability 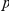, lifetime of loops 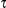), the center-of-mass distance is scaled with the mean radius of gyration 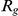 of the corresponding isolated chains. The radius of gyration is a measure of the typical size of a chromosome, i.e. the chromosome territory.

Evidently, the effective potential increases when approaching the two chromosomes, i.e. lowering the center-of-mass distance 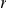. This result is expected, as the accessible conformational space becomes smaller the more the monomer clouds are in proximity. More importantly, we find that the effective potential 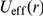 is pronouncedly stronger for chromosomes with a large average number of loops compared to linear chains. To assess whether these results are dependent on the chain length, and therefore the level of coarse-graining used, we have plotted the same results for chain lengths 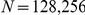 and 512 in Figure S1. Obviously, the dependence on chain length using the scaled center-of-mass distance is rather subtle, indicating that the level of coarse-graining used in simulations does not affect the results on the large scale. This is well-known to be true for self-avoiding walks and ring polymers, where the effective potential at full overlap adopts a constant value in the order of 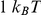 in the limit of infinite chain length. For the Dynamic Loop model, a comparison is more difficult, since the effective potential is also a non-trivial function of the looping probability 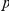. Importantly, the repulsive potential increases most strongly in the range where 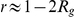, i.e. around the size of the chromosome territory, indicating a huge energy cost for a high degree of intermingling of chromosome territories.

**Figure 2:**
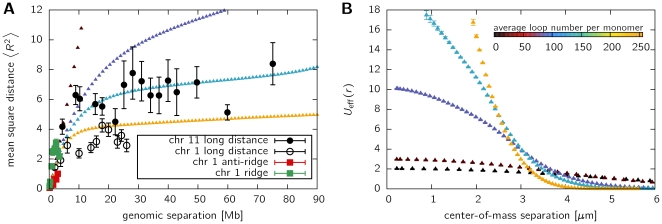
Mapping of coarse-grained polymers to physical parameters. Shown are results for a chain length of 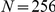 using different looping probabilities. Experimental data shows results from FISH measurements [19] on human chromosome 1 and 11. The chain is mapped to chromosome 11 by assuming one bead to comprise a 400kb-stretch of chromatin. Consistent with the experimental data, this is set equal to 480 nm. **A**. This panel shows the mean square distance in relation to genomic separation of model and experimental data to assess the quality of the mapping. **B**. The potential of mean force between two model chromosomes in relation to physical distance 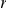 between the centers of mass. The effective potential strongly increases with increasing looping number at a separation of about 2–3 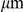, i.e. the size range of the assumed chromosome territories.

To demonstrate how the data can be mapped onto physical units, we use model polymers of chain length 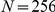. We set one coarse-grained bead to 400 kb in order to model a sufficiently long stretch in the size range of a typical chromosome. To determine the spatial extend of this stretch of chromatin, we employ long distance experimental data from human chromosome 11 [19]. In principal, such a mapping is always connected with a lot of uncertainy: The detailed Kuhn length is not known, disallowing for a precise mapping on the short scale; as chromatin is organized much more complex than a linear chain, other parameters (looping, binding, heterogeneity) enter the calculations. To obtain a simple mapping, we adjust the plateau level of the model polymers to that of experimental data. Figure 2A shows the results of the mapping using 177 nm for one lattice unit. Clearly, given such a coarse-graining approach, each polymer segment has to be considered as a bunch of chromatin, making it impossible to resolve the detailed interactions leading to the loop on a molecular basis. Importantly, the model displays well the leveling-off observed in experiments for intermediate looping probabilities (the cyan symbol ▴ corresponds to 131 loops on average). The effective potential 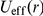 in units of 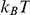 is displayed in Figure 2B. While the effective potential profile is rather flat for self-avoiding walks, the existence of loops leads to a strong increase in the potential at distances of about 2–3 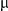
m, the region where the experimental data displays a leveling-off. Importantly, these results are independent of the looping lifetime (Figure S2).

**Figure 3:**
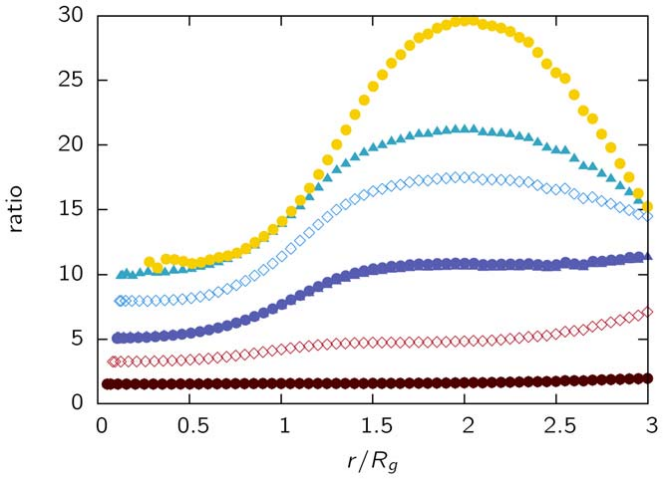
Ratio between the effective potential of looping polymers and linear chains. The data shows the ratio 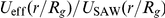 for chains of length 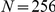 and different looping probabilities 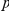. The data is plotted against the center-of-mass separation scaled by the radius of gyration 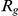 of isolated polymers. The figure symbols and color codes are the same as in Figure 2.

The quantitative increase in the effective potential of looping polymers over linear chains (self-avoiding walks) is shown in Figure 3. The factor 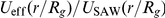 by which the effective potential of the model chromosomes is larger than that of the linear chain is plotted on the 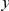-axis. Standard errors are in the size range of the symbols and therefore not displayed. Likewise, the abscissa shows the center-of-mass distance scaled by the radius of gyration 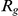. We find that the repulsive potential is stronger by more than one order of magnitude for chains in the parameter range where leveling off occurs.

### Looping polymers become aspherically elongated

Chromosomes, when brought in close proximity, not only reveal a strong repulsion between their centers of mass; besides this, their structural properties undergo significant changes. Here, we investigate how size and shape of a model chromosome changes in presence of a second one. Such effects play an important role inside the cell nucleus, as chromosomes are located in a complex environment being typically separated by only a few 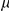
m; comparison to linear chains allows us to learn something about the effect of looping. The change in dimensionality is measured by the swelling factor 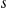, given by
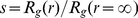
(1)Here, 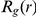 denotes the root mean squared radius of gyration for a chromosome being in a distance 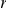 to a second one. 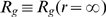 denotes the corresponding quantity for isolated chromosomes. In a recent study on topological effects between ring polymers [17] it has been shown that both linear chains as well as ring polymers swell when being brought together, the swelling factor being about 10% for rings and slightly smaller for linear chains.

**Figure 4:**
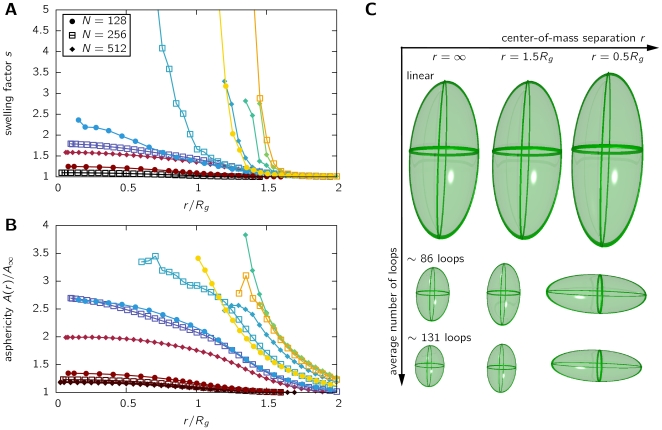
Structure of chromosomes being in proximity of a second one. A. The swelling factor 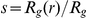 of the chromosome when being at a center-of-mass separation 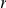 compared to the isolated case. Data is shown for different chain lengths 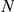 and looping probabilities 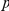. The symbols and colors used are the same as in Figure 1. B. The scaled asphericity 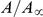 for model chromosomes in proximity. The same data is used as in panel A. C. Illustrations of the average gyration ellipsoids of the chromosomes. Shown is the change in shape and size of the gyration ellipsoids for linear chains (no loops), chromosomes with an average number of 86 loops (purple symbol ▴ in Figure 2B) and chromosomes with an average number of 131 loops (cyan symbol ▴ in Figure 2B).

While linear chains and ring polymers only show a mild swelling in the presence of a second chain, we find that model chromosomes swell enormously. Figure 4A displays 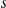 for different looping probabilities 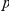, i.e. different values of the average number of loops, in relation to the scaled center-of-mass distance 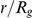. To demonstrate similar behaviour independent of the level of coarse-graining, results are shown for different chain length 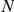. Swelling factors 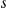 are strongly dependent on the average number of loops, increasing by a factor in the order of 2–10 for the range of looping probabilities where a leveling-off is observed (cf. also Figure 2A). In fact, 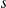 diverges for large loop numbers, indicating that the chains can not be approached closer than approx. 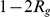.

The swelling of the chromosomes might suggest that they open up to create space for the monomers of the other chromosome, i.e. allow for intermingling. In the following, we will show that this is not the case, rather the contrary is observed. To achieve this, we investigate how the shape of the chromosomes changes when being close together. The asphericity 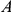 of the gyration ellipsoid has been established [15], [25] as a measure of shape, being zero for a spherically shaped polymer and unity for a rod-like polymer,
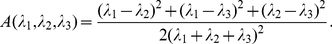
(2)Here, 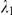, 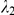 and 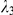 denote the eigenvalues of the gyration tensor. To highlight the changes in asphericity when approaching two chromosomes, we show the ratio 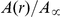 in Figure 4B, 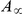 being the asphericity of an isolated model chromosome with the same parameters. While the change in asphericity for linear chains (self-avoiding walks, no loops) is rather small even at full overlap (about 20%), we find a pronounced aspherical deformation on our model chromosomes in the regime of looping probabilities that force a leveling-off in the mean square distance. Asphericity values increase by about 200–400% at genomic separations of 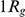, i.e. the typical size of the chromosome.

The changes in shape and dimension are visualized in Figure 4C. Shown are the average gyration ellispoids of three different model polymers: (i) linear chains (self-avoiding walks, 0 loops), (ii) chromosomes with 86 loops on average (purple symbol ▴ in Figure 2B) and chromosomes with 131 loops on average (cyan symbol ▴ in Figure 2B). For each set of model parameters, the ellipsoids are displayed for three different center-of-mass distances: isolated chains (infinite CM distance), 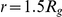 and 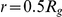. We find that isolated linear chains require a huge amount of space, while looping polymers are pronouncedly smaller and more spherical. When being in contact with a second chromosome, the shape of self-avoiding walks changes only slightly, while chromosomes with loops become markedly aspherical compared to their isolated shape.

These findings indicate that chromosomes, similar to ring polymers, do not swell in order to create space for the second chromosome. On the contrary, they swell to avoid each other by elongating in such a way that the overlapping is minimized. This is achieved by aligning the highly aspherical gyration tensors in a parallel way when close together.

### Looping polymers avoid intermingling

**Figure 5:**
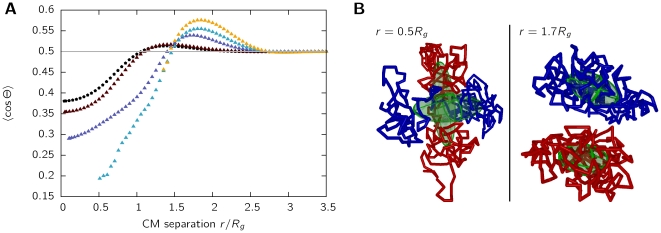
Mutual alignment of the gyration ellipsoids. A. The average angle 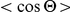 is shown in dependency of the center-of-mass separation 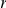 for chains of length 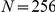. Black symbols correspond to a linear chain (SAW), colored symbols to chromosomes with loops, the color coding being the same as in Figure 2. Error bars are smaller than the symbol size and therefore not shown. The grey line corresponds to a random orientation of the gyration ellipsoids, showing that chromosomes with loops induce a non-random mutual alignment. B. Two chromosomes with a fixed center-of-mass separation 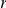. The left-hand image shows chromosomes in the regime where perpendicular alignment is observed (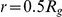), the right-hand image displays chromosomes with a center-of-mass separation of 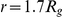.

To answer the question whether chromosomes swell to create space for each other or if they rather try to avoid each other, the mutual alignment of the polymers is studied. An established measure for the mutual alignment is given by the average angle 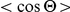 between the gyration tensors largest principal axes [15], [17]. In case of the chromosomes being adjusted independently of each other, the average angle would adopt the value of 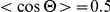 [15]. This value arises by averaging over two randomly oriented orientations in three-dimensional space. Deviations from this value indicate a tendency of the polymers to align in a certain non-random way with respect to each other. Figure 5 displays results for linear chains (black symbols) and chromosomes with loops in relation to the center-of-mass distance for chains of length 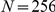. The symbol and color code used is the same as in Figure 2. We find that chromosomes display a pronouncedly stronger tendency to align perpendicular at short center-of-mass separations than linear chains or ring polymers (cf. 5A). Similarly, a slightly parallel alignment can be found at intermediate distances.

These findings might be explained by a tendency of the chromosomes to minimize the overlap area. When the distance between chromosomes is lowered to values in the order of the size of the chromosome, they start to feel the presence of the second chromosome. Thus, the space of accessible conformations is reduced and the chromosome stretches in a direction perpendicular to the center-of-mass axis. However, when the chromosomes are forced even closer together, nearly overlapping completely, the observed perpendicular alignment together with the strong elongation minimizes the volume shared by both chains (Figure 5B).

**Figure 6:**
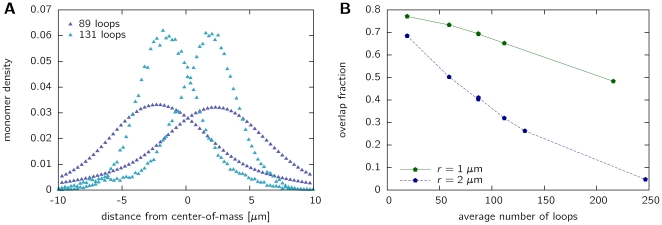
Segregation of chromosomes with loops. A. This panel shows the monomer density distribution projected onto the line connecting the centers of mass of both chromosomes. A distance of 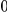 indicates the point halfway between the centers of mass, which are in this example separated by 1 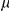
m. Results are shown for chain lengths of 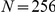 using the mapping of Figure 2. B. The degree of intermingling is measured by the overlap area of the monomer distributions from both chromosomes in panel A. The overlap fraction is given for two different center-of-mass separations: (i) 1 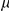
m and (ii) 2 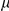
m. Results show that the overlap fraction, i.e. the degree of intermingling decreases strongly with the average number of loops in the system.

To quantitatively assess the amount of intermingling between the model chromosomes in dependency of the average number of loops, we project the monomer positions to the line connecting the centers of mass of both chromosomes. Thus, a density distribution can be obtained as shown in Figure 6A. Here, chromosomes with a coarse-grained length of 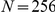 have been simulated. Mapping is done according to the procedure described above. Results are shown for different looping probabilities and a fixed center-of-mass distance 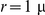
m. The average number of loops being 0 (linear case, black symbol), 86 (purble symbol ▴) and 131 (purple symbol ▴). Clearly, introducing loops in the chromatin structure results in more compact polymers, the monomers being distributed closer around the centers of mass. We determine the overlap fraction by integrating the overlap area between the distributions of both chromosomes. The results are shown in Figure 6B using two different center-of-mass separations 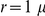
m and 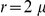
m. For a center-of-mass distance of 2 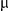
m, being comparable to the size of chromosomal regions [19], we find that the overlap fraction decreases strongly from about 0.7 down to less than 0.1 for large loop numbers. Interestingly, in the range where leveling-off occurs, overlap fractions are in the range of 20–30%. These values are, however, an overestimate, as the projection procedure does not capture segregation in the direction perpendicular to the line connecting the centers of mass. Although not directly comparable to experimental results, these values are in the size range of experimental data from FISH cryo sections, where an overlap volume of 20% has been observed [26].

## Discussion

In this paper, we have analyzed the effect of loops on the repulsive interactions between polymers. As a measure for these interactions we applied the theory of effective potentials, where monomeric degrees of freedom in the partition sum are traced out. The resulting effective potential 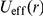 gives the interaction between both polymers in dependence of their center-of-mass distance 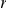. 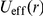 has been determined for self-avoiding walks in recent decades [24] and for ring polymers [17]. Both linear polymers and rings display a repulsive interaction at full overlap (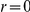), asymptotically converging to a finite value in the order of 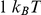 in the limit of large chain lengths. Here, we applied the concept of effective interactions to the Dynamic Loop model, which has been proposed as a model for chromatin organization in Ref. [18].

The major finding of this study is that introducing dynamic loops in the structure of chromatin results in a strong increase of the repulsive interactions by about one order of magnitude (Figure 1). Using a mapping to physical units based on recent FISH experiments, we found that the repulsive forces are strongest at center-of-mass separations of 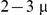
m, i.e. the size of the chromosome territory (Figure 2). These observations indicate that chromatin looping plays a dominant role in the entropy-driven segregation of chromosomes.

Moreover, we found that the existence of loops introduces strong changes in the size and shape properties of the chromosomes. Indeed, when being brought close together, looping polymers swell and become pronouncedly aspherical, the observed effect being multi-fold larger than for linear chains or ring polymers. Chromosomes in proximity display a highly non-random orientation of their gyration ellipsoids. Increasing the number of loops leads to a significant decrease in the overlap fraction.

Clearly, the modelling approach pursued here is simplified with regard to the cellular system. In reality, we have a dense system of chromosomes with a chromatin content of about 10%. It remains to be studied in further publications, whether bringing multiple chromosomes together leads to even stronger effects. Here, for ease of simplicity, we focussed on a two-chromosome-system because it allows best to highlight pairwise interactions between chromosomes and the effect of loops while keeping simulational effort reasonable. Studying the impact of multiple chromosome in a dynamic system the future might also shed light on the question whether the proximity between chromosomes reversly might affect looping probabilty. This, however, is not possible in the framework of the Dynamic Loop Model as presented here because it takes the looping probability as an input parameter.

Our findings indicate that chromatin loops not only play an important role in transcriptional regulation. Rather, they help to impose a certain state of order and segregation. Thus, loops seem to constitute a highly efficient regulatory mechanism concerning gene regulation as well as chromatin compartmentalization. The Dynamic Loop model used in this study refrains from assuming active driving mechanisms for loop formation, rather loops form by diffusional motion, minimizing the energy cost.

## Materials and Methods

### Computer simulations of chromosomes

Model chromosomes are simulated using the Dynamic Loop model introduced in Ref. [18]. The Dynamic Loop model is implemented employing lattice Monte Carlo simulations [27] in order to simplify the handling of excluded volume. Calculation of excluded volume interactions thereby is reduced to checking whether one lattice site is already occupied or not. To allow for more flexibility than a simple local-move algorithm on a cubic lattice, we employ the bond-fluctuation method introduced by Carmesin [28] allowing 108 different bond vectors; the length of a bond can take the values 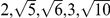 [29]. The bond-fluctuation model is especially suited for dense and compact systems where a lattice algorithm would no longer be feasible due to high rejection rates during the Monte Carlo process. The simulation method applied fulfills the following important features: (i) it produces unbiased results, i.e. each possible conformation out of the ensemble is sampled with equal probability, (ii) it takes into account excluded volume interactions, i.e. two monomers are not allowed to occupy the same region in space and (iii) using some restrictions on the moves and bond vectors it ensures that no bond crossings can occur during a Monte Carlo step, i.e. it preserves the topological state of the conformation. The algorithm conducts only local moves in order to resemble the dynamics of real polymers [28]. Using a coarse-grained lattice approach is reasonable as we are only interested in features of looping chromatin independent on local structure. Coarse-graining allows us to abstract from the complex environment and highlight the main driving forces and effects of chromatin folding.

We perform simulations of isolated chromosomes with coarse-grained lengths ranging from 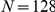 to 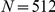. Chromosomes are initially equilibrated as self-avoiding walks using local moves of a monomer to one of the nearest neighbors on the lattice. After the initial equilibration steps, the Monte Carlo algorithm allows for the formation of loops. After each Monte Carlo trial move, one monomer is selected at random. It is then checked whether another monomer on the same chain is in the neighborhood, i.e. co-localized. The co-localization condition is fulfilled whenever the distance between the monomers is less than 3 lattice units. If the two monomers are co-localized, then a loop is formed with a certain probability 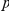. If the loop 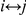 is created, it is assigned a certain lifetime 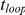 which is drawn from a Poissonian distribution
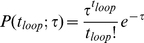
(3)where the parameter 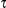 determines the average lifetime of the loops. The lifetime of the loops is chosen here to be
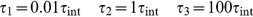
(4)As it has been shown [17] that the chosen lifetime 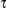 does not strongly influence equilibrium properties, parts of the analysis presented here are restricted to the choice 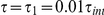.

Since subsequent conformations in the Markov chain created by the Monte Carlo algorithm are highly correlated, one has to perform a certain number of Monte Carlo steps to obtain two independent conformations. For each set of parameters (chain length 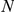, looping probability 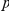 and lifetime of loops 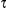) we determine the autocorrelation function 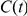 (see e.g. Ref. [30]) of the squared radius of gyration 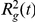. The integrated autocorrelation time 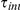 is then determined using the windowing method introduced by Sokal [31].

### Calculation of the effective potential

To analyze the strength of the repulsive interactions, the potential acting between the chromosomes' centers of mass is determined using the method introduced by Dautenhahn and Hall [24]. In short, two equilibrated isolated chromosome conformations are selected and shifted such that the distance between their centers of mass equals 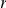. If the excluded volume condition is satisfied, i.e. no lattice site is occupied by more than one bond, the conformation is accepted, otherwise it is rejected. The fraction of accepted conformations 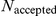 to the total number 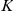 of trial conformations defines the effective potential at distance 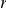,
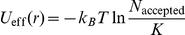
From the set of accepted two-chain conformations, the conformational properties can be calculated.

## Supporting Information

Figure S1 The effective potential *U_eff_(r)* between the centers of mass of looping polymers. Simulations have been conducted using the DL model for different chain lengths *N* and different looping probabilities *p*. The average number of loops per monomer resulting from the parameter *p* is indicated by the color bar. Data is scaled with the radius of gyration of isolated polymer chains to allow comparison between different parameters sets. The effective potential increases strongly with looping probability *p*, but is basically independent of the level of coarse-graining (i.e., the chain length) used.
(0.14 MB PDF)

Figure S2 Mapping of coarse-grained polymers to physical parameters. Shown are results for a chain length of *N = 256* using different looping probabilities. Experimental data shows results from FISH measurements on human chromosomes 1 and 11. The chain is mapped to chromosome 11 by assuming one bead to comprise a 400-kb stretch of chromatin. Consistent with the experimental data, this is set equal to 480nm. Different symbols indicate different looping lifetimes (Black triangle: τ = 0.01\τ_int_; Diamond: τ = τ_int_; Bullet: τ = 100\τ_int_, see Materials and Methods). (A) This panel shows the mean square distance in relation to genomic separation of model and experimental data to assess the quality of the mapping. (B) The potential of mean force between two model chromosomes in relation to physical distance *r* between the centers of mass. The effective potential strongly increases with increasing looping number at a separation of about 2–3 µm, i.e., the size range of the assumed chromosome territories.
(0.15 MB PDF)
