# Supplementary material for: Repulsive Forces Between Looping Chromosomes Induce Entropy-Driven Segregation
Source: PLoS One. 2011 Jan 4;6(1):e14428. doi: 10.1371/journal.pone.0014428 (PMC3014947; doi:10.1371/journal.pone.0014428)

Figure S1

The effective potential $U_{\text{eff}}(r)$ between the centers of mass of looping polymers. Simulations have been conducted using the DL model for different chain lengths N and different looping probabilities p. The average number of loops per monomer resulting from the parameter p is indicated by the color bar. Data is scaled with the radius of gyration of isolated polymer chains to allow comparison between different parameters sets. The effective potential increases strongly with looping probability p, but is basically independent of the level of coarse-graining, i.e. the chain length, used

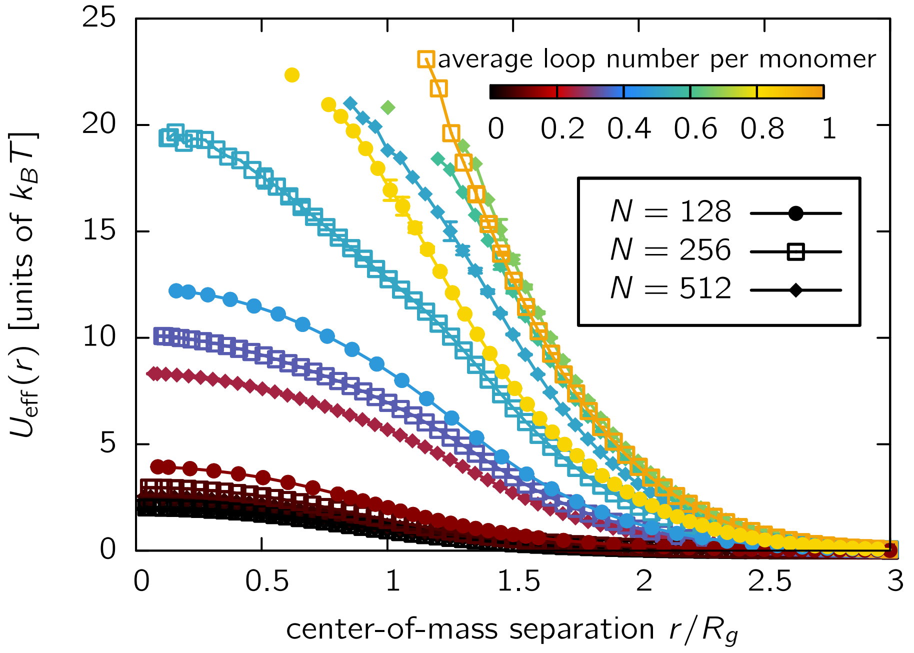

Supplement: Figure S1 — The effective potential Ueff(r) between the centers of mass of looping polymers. Simulations have been conducted using the DL model for different chain lengths N and different looping probabilities p. The average number of loops per monomer resulting from the parameter p is indicated by the color bar. Data is scaled with the radius of gyration of isolated polymer chains to allow comparison between different parameters sets. The effective potential increases strongly with looping probability p, but is basically independent of the level of coarse-graining (i.e., the chain length) used. (0.14 MB PDF) [file pone.0014428.s001.pdf]
