# Supplementary material for: Repulsive Forces Between Looping Chromosomes Induce Entropy-Driven Segregation
Source: PLoS One. 2011 Jan 4;6(1):e14428. doi: 10.1371/journal.pone.0014428 (PMC3014947; doi:10.1371/journal.pone.0014428)

Figure S2

Mapping of coarse-grained polymers to physical parameters. Shown are results for a chain length of $N = 256$ using different looping probabilities. Experimental data shows results from FISH measurements on human chromosome 1 and 11. The chain is mapped to chromosome 11 by assuming one bead to comprise a 400kb-stretch of chromatin. Consistent with the experimental data, this is set equal to 480 nm. Different symbols indicate different looping lifetimes (▲: $\tau = 0.01\tau_{int}$; ◊: $\tau = \tau_{int}$; •: $\tau = 100\tau_{int}$, see Materials & Methods). **A.** This panel shows the mean square distance in relation to genomic separation of model and experimental data to assess the quality of the mapping. **B.** The potential of mean force between two model chromosomes in relation to physical distance r between the centers of mass. The effective potential strongly increases with increasing looping number at a separation of about 2-3 μm, i.e. the size range of the assumed chromosome territories.

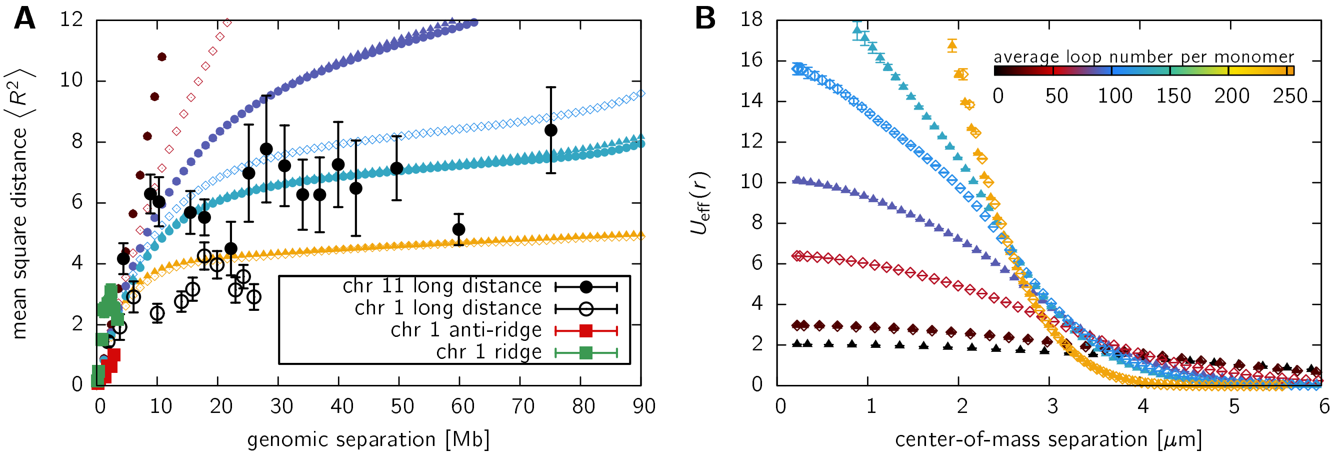

Supplement: Figure S2 — Mapping of coarse-grained polymers to physical parameters. Shown are results for a chain length of N = 256 using different looping probabilities. Experimental data shows results from FISH measurements on human chromosomes 1 and 11. The chain is mapped to chromosome 11 by assuming one bead to comprise a 400-kb stretch of chromatin. Consistent with the experimental data, this is set equal to 480nm. Different symbols indicate different looping lifetimes (Black triangle: τ = 0.01\τint; Diamond: τ = τint; Bullet: τ = 100\τint, see Materials and Methods). (A) This panel shows the mean square distance in relation to genomic separation of model and experimental data to assess the quality of the mapping. (B) The potential of mean force between two model chromosomes in relation to physical distance r between the centers of mass. The effective potential strongly increases with increasing looping number at a separation of about 2–3 µm, i.e., the size range of the assumed chromosome territories. (0.15 MB PDF) [file pone.0014428.s002.pdf]
